# Ultrasound supports clinical decision-making in determining the Sanders’ skeletal maturity score of the hand

**DOI:** 10.1007/s00264-024-06184-7

**Published:** 2024-04-24

**Authors:** Ferdinand Wagner, Stefanie König, Quirin Johannes Wuermeling, Alexandra Sitzberger, Marco Paolini, Annabelle Weigert, Michael Lauseker, Felix Endres, Julia Schneller, Jochen Hubertus, Boris Michael Holzapfel, Christof Birkenmaier, Christian Max Ziegler

**Affiliations:** 1 Department of Orthopaedics and Trauma Surgery, Musculoskeletal University Center Munich (MUM), LMU University Hospital, Ludwig-Maximilians-Universität München, Marchioninistrasse 15, 81377 Munich, Germany; 2 Department of Pediatric Surgery, Dr. Von Hauner Children’s Hospital, Ludwig-Maximilians-Universität München, Lindwurmstraße 4, 80336 Munich, Germany; 3 Department of Pediatric Neurology and Developmental Medicine, LMU Hospital, LMU Center for Children With Medical Complexity, Dr. Von Hauner Children’s Hospital, Ludwig-Maximilians-Universität (LMU), Lindwurmstraße 4, 80336 Munich, Germany; 4 Department of Radiology, University Hospital, LMU University Hospital, Ludwig-Maximilians-Universitäty München, Marchioninistrasse 15, 81377 Munich, Germany; 5 Institute for Medical Information Processing, Biometry, and Epidemiology, Faculty of Medicine, Ludwig-Maximilians-Universität München, Marchioninistrasse 15, 81377 Munich, Germany; 6 Department of Pediatric Surgery, Marien Hospital Witten, Ruhr-University Bochum, Marienplatz 2, 58452 Witten, Germany; 7 Artemed Klinikum München Süd, Am Isarkanal 30, 81379 Munich, Germany; 8 Institute of Health and Biomedical Innovation, Queensland University of Technology (QUT), 60 Musk Ave, Kelvin Grove, Brisbane, QLD 4059 Australia

**Keywords:** Ultrasound, Skeleton, Growth prognosis, Sanders Maturity score

## Abstract

**Purpose:**

The Sanders Scoring System has revolutionized the way we assess the remaining growth potential of the skeleton. However, because it involves radiation exposure, it must be used with caution in children. The purpose of the study was to evaluate whether the Sanders skeletal maturity score (SMS) could be accurately determined using ultrasound (U).

**Methods:**

We took radiographs (*R*) of the hand and performed *U* of the thumb and index finger in 115 patients between six and 19 years of age who were undergoing treatment for scoliosis or limb deformities. Paediatric orthopaedic surgeons, a paediatrician, and a paediatric radiologist were evaluated the blinded images. Those classified images are based on the SMS and the Thumb Ossification Composite Index (TOCI).

**Results:**

Intrarater reliability was high for SMS and slightly weaker for TOCI, but still significant. Interrater reliability was clear for *R* and weaker for *U* in both staging systems. Ultimately, SMS 3 and 7 achieved the highest percentage of concordance (*P*) of 71.7% and 66.0%, respectively, when U was performed. Combining the clinically relevant groups of SMS 3&4 and SMS 7&8 also significantly increased peak scores (SMS 3 and 4 *P* = 76.7%; SMS 7 and 8 *P* = 79.7%). The probabilities of peak scores were significantly weaker when the TOCI score was examined.

**Conclusion:**

Our study shows that *U* can be used effectively especially to measure stages 3 and 4 and stages 7 and 8 of SMS. The *U* method is easy to use and therefore may offer advantages in clinical practice without the need for radiation exposure.

## Introduction

The assessment of growth prognosis is crucial for the treatment of scoliosis and growth guidance measures such as temporary epiphysiodesis [1–3]. For most physicians, the bone age determination atlas of Greulich and Pyle is still a standard method, but it is very cumbersome to implement [4]. Therefore, Sanders et al. developed a score that predicts the progression of the last growth period well and is easy to apply without the use of a detailed atlas book. However, both methods require an x-ray of the non-dominant hand [5]. Other methods like the evaluation of the growth plates or apophyses situated at the pelvis, the thumb, the proximal humerus, or the calcaneus have been described [6–9]. Hung et al. introduced the Thumb Ossification Composite Index (TOCI) using only a radiograph of the thumb.

Although in principle this does not imply a high radiation exposure, recent high-quality studies have shown that in a growing organism even low radiation doses carry a higher risk of malignancy than in adulthood. Therefore, the ALARA principle (“As Low As Reasonably Achievable”) should always be followed [10–12]. Especially in children, sonography has therefore proven to be an invaluable radiation-free diagnostic tool [13–17]. These studies investigated growth plates or apophyses that were either difficult to access or poorly documented for predicting growth. Mentzel et al. and Utczas et al. created an ultrasound (U) device to assess skeletal maturity, although it is not widely available and does not utilize a standard U system [15, 17].

Therefore, we questioned whether U is suitable to identify the Sanders stages of the hand or the TOCI score of the thumb with special focus on the stages relevant for therapy. Sanders stages 3 and 4 (S3 and 4) are crucial for deciding if to start brace therapy for scoliosis, while stages 7 and 8 (S7 and S8) are significant for determining when to stop brace therapy or for deciding against epiphysiodesis in cases of limb deformities.

## Methods

### Patient acquisition

We enrolled children aged six to 19 years who were treated in our clinic for scoliosis, leg length discrepancy, or leg axis deformities. In these patients, the growth prognosis is crucial for the treatment decision. Children with a bone metabolism disorder such as rickets, genetic or syndromal disease of the skeletal system, and patients with active endocrine disease affecting the skeletal system were excluded.

From October 2020 to December 2021, 136 subjects who underwent radiographic examination (R) of bone age and met the study criteria underwent additional U in our department.

We obtained written informed consent from the patients and legal guardians after a verbal explanation of the aim of the study and its modalities. The study was approved by the local ethics committee of the Ludwig Maximilians University of Munich (approval number 20-0780) and was conducted in accordance with the Declaration of Helsinki.

### Ultrasound method

To ensure a standardized examination, *U* was performed by two paediatric orthopaedic surgeons (F.W. and C.M.Z) or by a graduate student (Q.W. and S.K.) under close supervision of the former. We used a single sonographic device for 113 patients (Philips Affiniti 50, Philips Ultrasound Inc., WA, USA) and a newer device (General Electric Venue R3, GE Medical Systems SCS, France) for the remaining 23. The quality of images was equal with both devices.

**Fig. 1:**
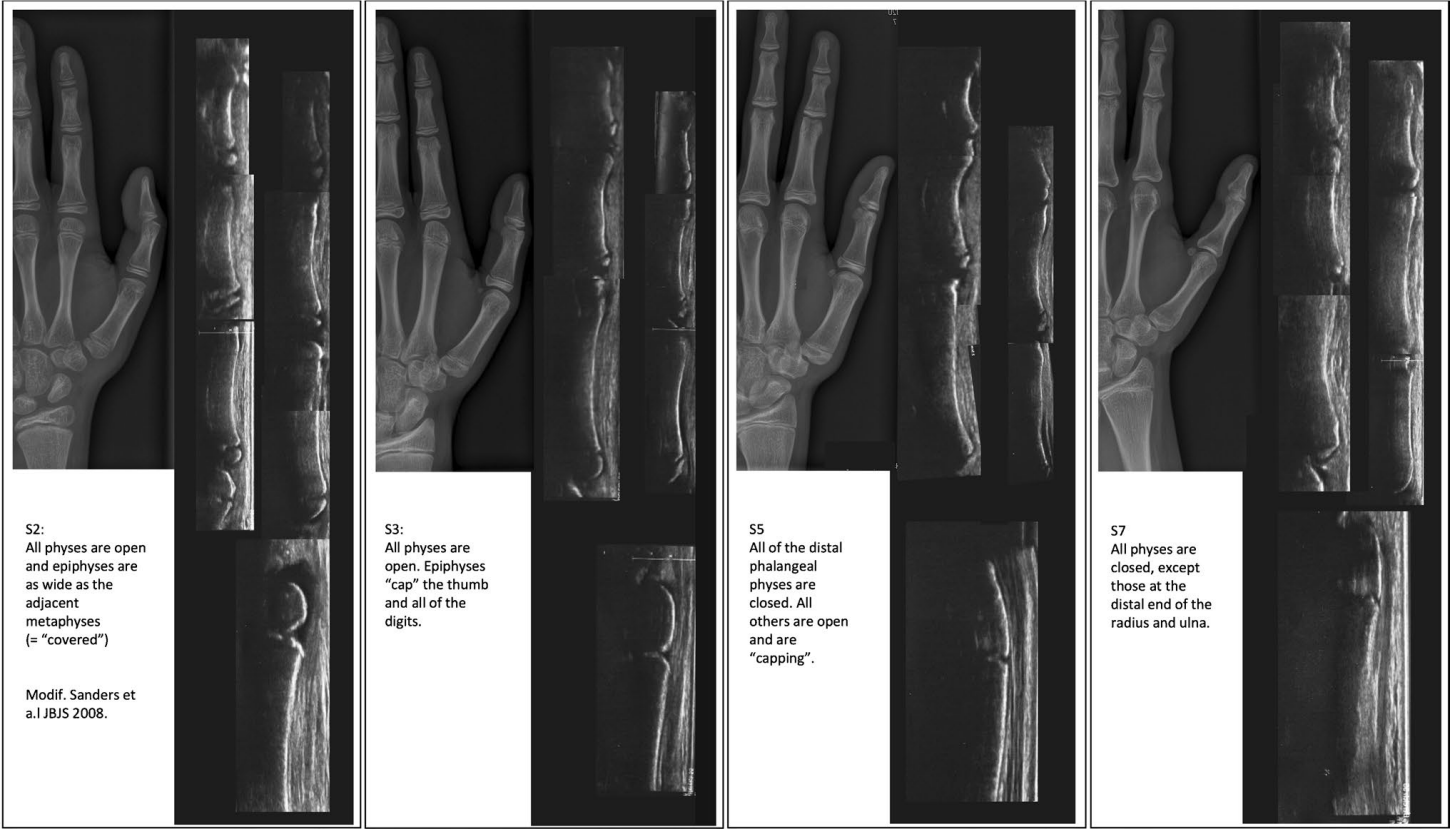
Examples for ultrasound performed of the thumb and index finger of the hand with Sanders stages 2, 3 5, and 7 with the corresponding X-rays of the hand

A total of seven longitudinal sonographic sections of the hand were obtained. We acquired lateral radial images of the index finger (distal, middle, and proximal phalanges), thumb (distal and proximal phalanges and the 1st metacarpal), and a lateral view of the distal radial growth plate (Fig. 1).

### Image analysis

The radiographs and the sonographs were presented to the raters in separate files. Scores were recorded on two separate pseudonymized score sheets by a designated orthopaedic specialist, a paediatric orthopaedic attending, a neuropaediatrician, a paediatric radiologist, and a graduate student. To determine intrarater reliability, all data were scored twice at least two weeks apart by three observers (randomly chosen).

We scored the image quality of both *R* and *U* images from 1 to 3 points. One point was awarded for good tissue contrast, one for good hand/finger position, and one for no motion (poor, acceptable, and excellent).

### Statistics

For statistical analysis, we used SPSS (version 23, IBM, Armok, New York). Descriptive statistics were generated using absolute and relative frequency cross-tabulations. Reliability was measured using Fleiss’ and Cohen’s Kappa coefficients. The Fleiss *K* coefficient was calculated for the total cohort of five observers. The Cohen *K* coefficient was calculated for intrarater reliability for each of three observers. One-sided 95% confidence intervals were estimated for the percentage of convergence.

## Results

### Demographics

**Fig. 2:**
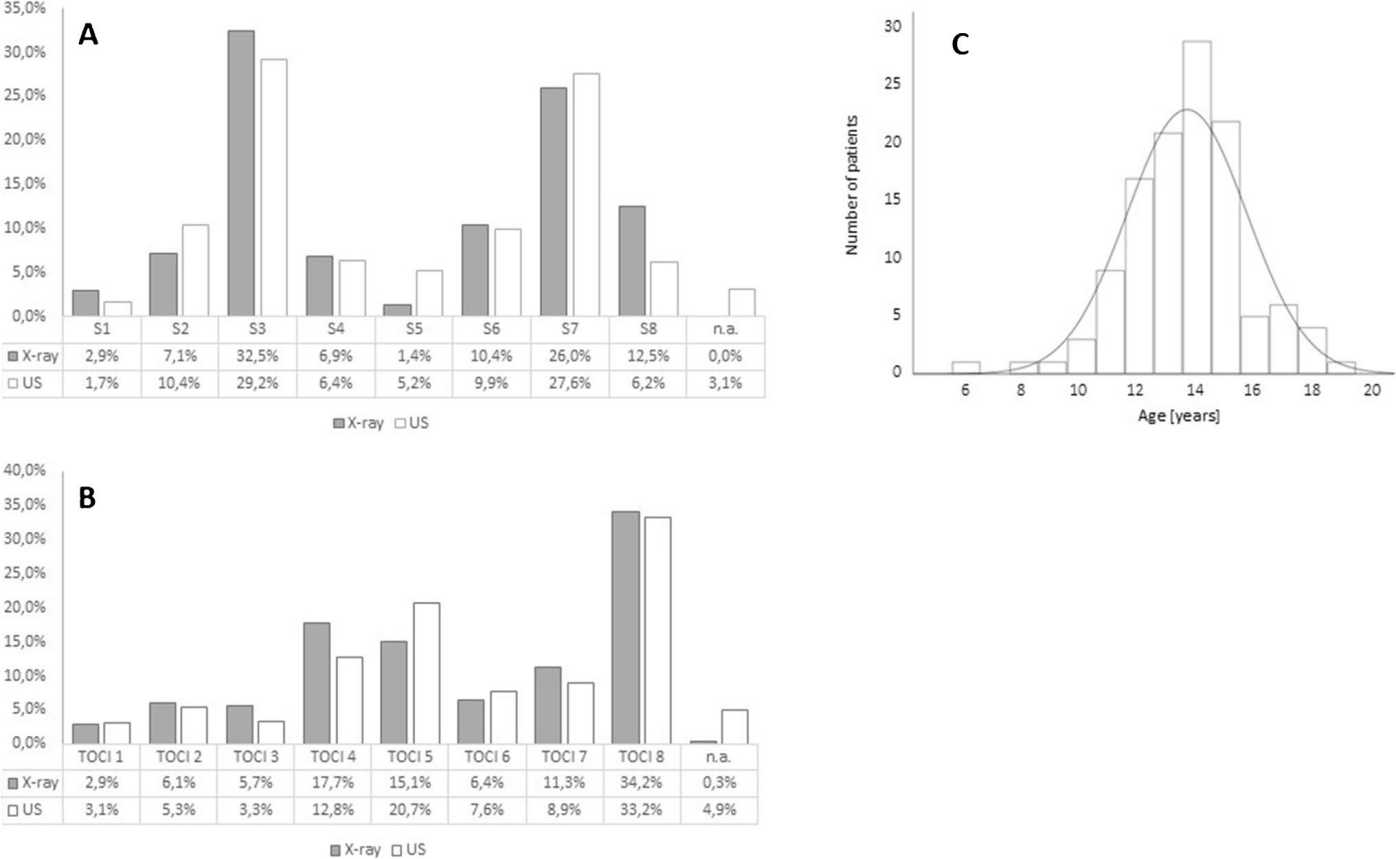
Percentage of all levels (**A** SMS and **B** TOCI) that were rated by the 5 different raters. The last line on the right shows the percentage of U-scans that could not be rated due to poor quality (n.a. = not applicable). **C** Age distribution of analyzed patients with a gaussian distribution curve

In total, we studied a patient population of 136 children who met the inclusion criteria. Of this cohort, 21 children (15.4%) dropped out due to either incomplete imaging or insufficient image quality (less than 2 points of either *U* or *R*), resulting in an evaluable number of 115 children. Eighty-five percent of patients were between 12 and 17 years of age (mean age 13.7 years, StDev ± 2.1). The gender distribution was nearly 1:1, and all investigators evaluated all 115 image pairs (*R* and the corresponding *U* image). Figure 2 shows the relative frequencies for the Sanders and TOCI scores. Most frequent scores were S3 (*R* = 32.5%; *U* = 29.2%), S7 (*R* = 26.0%; *U* = 27.6%), T5 (*R* = 15.1%; *U* = 20.7%), and T8 (*R* = 34.2%; *U* = 33.2%).

### Interrater reliability

As shown in Table 1, there was clear interobserver agreement for both Sanders and TOCI for R images. Weaker but almost clear agreement was found for SMS determined by *U* (*k* = 0.381).

**Table 1:** Interrater reliability for SMS and TOCI scores analyzed via Fleiss’ Kappa coefficient (*k*)

Interrater reliability					
Mean	*R* (Fleiss’ Kappa)	*U* (Fleiss’ Kappa)			
SMS	0.592 *	*0.381*			
TOCI	0.440 *	*0.333*			
Concordance of ultrasound and X-ray					
SMS	*Rater 1*	*Rater 2*	*Rater 3*	*Rater 4*	*Rater 5*
Percentage of concordance (one-sided 95% confidence interval)	*P* = *70.4%* *(95% CI [63.1%; 100%])*	*P* = *53.9%* *(95% CI [46.3%; 100%])*	*P* = *33.0%* *(95% CI [26.2%; 100%])*	*P* = *70.4%* *(95% CI [63.1%; 100%])*	*P* = *62.6%* *(95% CI [55.0%; 100%])*
TOCI	*Rater 1*	*Rater 2*	*Rater 3*	Rater 4	*Rater 5*
Percentage of concordance (one-sided 95% confidence interval)	*P* = *68.7%* *(95% CI [63.1%; 100%])*	*P* = *40.0%* *(95% CI [32.7%; 100%])*	*P* = *37.4%* *(95% CI [37.4%; 100%])*	*P* = *48.7% (95% CI [41.1%; 100%])*	*P* = *67.0%* *(95% CI [59.5%; 100%])*

The following interpretation was applied: *k* < 0.1: no match; 0.1 < *k* ≤ 0.4: weak agreement; *0.4 < *k* ≤ 0.6: clear agreement; **0.6 < *k*≦0.8; strong agreement; ***0.81 < *k* ≤ 1: (almost) complete agreement

### Intrarater reliability

Three of the five raters rated the images twice. For these raters, the first rating was used for statistical analysis. When analyzing the intrarater reliability of the three examiners mentioned above, there was strong to almost complete agreement on both evaluation sheets, for both *R* and *U* (see Table 2). Only examiner 2 was below the threshold of 0.4 for TOCI on the radiographs.

**Table 2:** Intrarater reliability for SMS and TOCI scores analyzed via Cohen’s Kappa (*k*)

Intrarater reliability					
SMS	Rater 1	Rater 2	Rater 3	Mean	StDev
*R* (*k*)	0.842***	0.627**	0.929***	0.799 **	± *0.127*
*U* (*k*)	0.654**	0.595**	0.649**	0.633 **	± *0.027*
TOCI	Rater 1	Rater 2	Rater 3	Mean	StDev
*R* (*k*)	0.731**	*0.328*	0.854***	0.638 **	± 0.225
*U* (*k*)	0.653**	0.478*	0.667**	0.599 *	± 0.086

For both, the following interpretation was applied: *k* < 0.1: no match; 0.1 < *k* ≤ 0.4: weak agreement; *0.4 < k ≤ 0.6: clear agreement; **0.6 < *k*≦0.8; strong agreement; ***0.81 < *k* ≤ 1: (almost) complete agreement

### Overall intermodality agreement of scores (R vs. U)

**Fig. 3:**
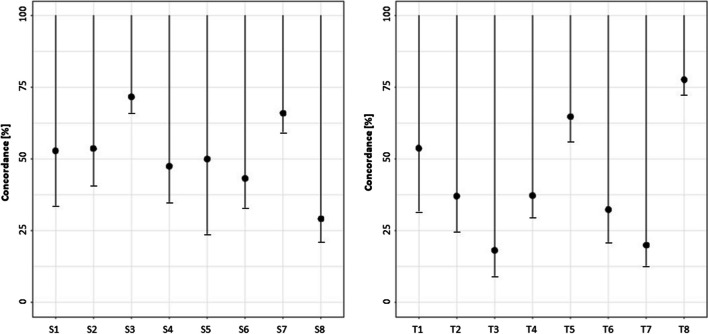
One-sided confidence intervals (CI) for the percentage of concordance in different Sanders and TOCI stages. Black dots indicate concordance; lower bars depict the lower bound of CI

Sanders 3 and 7 achieved the highest concordance with an estimated percentage of 71.7% and 66.0%. In comparison, Sanders 5 did not show high agreement (see Fig. 3 and Table 3). TOCI 1, 5, and 8 had the highest point probabilities at 53.8%, 64.8%, and 77.7%, respectively. The other TOCI stages did not show a high agreement (between 18 and 37%).

**Table 3:** Percentage of concordance (*P*) and 95%-confidence intervals (CI) for the different SMS and TOCI stages

Sanders	Percentage of concordance (P); one-sided confidence interval (CI)
SMS 1	*P* = ***52.9%****; CI [33.7%; 100%]*
SMS 2	*P* = ***53.7%****; CI [40.9%; 100%]*
SMS 3	*P* = ***71.7%****; CI [66.0%; 100%]*
SMS 4	*P* = ***47.5%****; CI [34.9%; 100%]*
SMS 5	*P* = ***50.0%****; CI [23.9%; 100%]*
SMS 6	*P* = ***43.3%****; CI [33.2%; 100%]*
SMS 7	*P* = ***66.0%****; CI [59.4%; 100%]*
SMS 8	*P* = ***29.2%****; CI [21.1%; 100%]*
TOCI	
TOCI 1	*P* = ***53.8%****; CI [32.0%; 100%]*
TOCI 2	*P* = ***37.1%****; CI [24.8%; 100%]*
TOCI 3	*P* = ***18.2%****; CI [9.3%; 100%]*
TOCI 4	*P* = ***37.3%****; CI [29.7%; 100%]*
TOCI 5	*P* = ***64.8%****; CI [56.3%; 100%]*
TOCI 6	*P* = ***32.4%****; CI [21.0%; 100%]*
TOCI 7	*P* = ***20.0%****; CI [12.9%; 100%]*
TOCI 8	*P* = ***77.7%****; CI [72.5%; 100%]*
TOCI 9	*Statistically not evaluable due to low case number*

*n* describes the numbers of images measured for *R* and *U*. [..] describes the lower and the upper bounds of CI

**Fig. 4:**
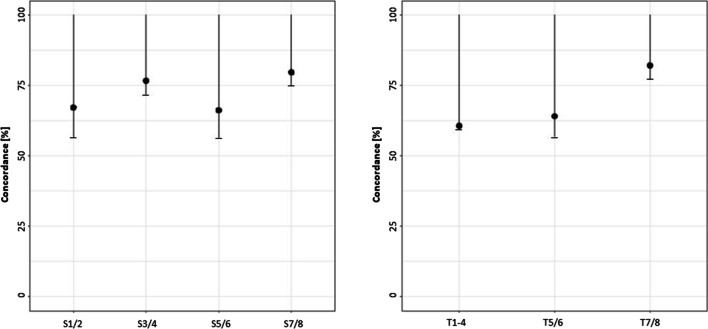
One-sided confidence intervals (CI) for the percentage of concordance in different summarized Sanders and TOCI stages. Black dots indicate concordance; lower bars depict the lower bound of CI

For simplification and better reflection of the clinically relevant groups, the 8 levels of Sanders and TOCI were summarized in four and three categories respectively. The concordance—with 95% confidence interval—is shown in Table 4 and Fig. 4. We found a lower concordance for TOCI compared to Sanders. Categories 2 (SMS 3/4) and 4 (SMS 7/8) in Sanders and Category 3 (TOCI 7/8)in TOCI showed the greatest accuracy (*P* = 76.7%; SMS 3/4 and *P* = 79.7%; SMS7/8 and *P* = 81.3%; TOCI 7/8).

**Table 4:** Condensed SMS and TOCI classifications with the percentage of concordance (P) and confidence intervals (CI)

Sanders		Percentage of concordance (P); one-sided confidence interval (CI)
Category 1	SMS 1/2	*P* = **67.2%**; CI [56.6%; 100%]
Category 2	SMS 3/4	*P* = **76.7%**; CI [71.8%; 100%]
Category 3	SMS 5/6	*P* = **66.2%**; CI [56.4%; 100%]
Category 4	SMS 7/8	*P* = **79.7%**; CI [75.0%; 100%]
TOCI		
Category 1	TOCI 1/2/3/4	*P* = **60.7%**; CI [53.4%; 100%]
Category 2	TOCI 5/6	*P* = **64.1%**; CI [56.9%; 100%]
Category 3	TOCI 7/8	*P* = **82.1%**; CI [77.1%; 100%]

[..] describes the lower and the upper bounds of CI

## Discussion

Growth prediction in early adolescence is of critical diagnostic importance in many musculoskeletal conditions that require monitoring until skeletal maturity and can be treated with surgical or conservative growth guidance [2, 18, 19]. Unfortunately, current methods still rely on radiation-driven diagnostics such as radiography of the hand or pelvis. Since the ALARA principle is always binding, we investigated whether the SMS and the TOCI can be performed with sufficient accuracy using U [11, 12]. In this study, we provide fundamental evidence that U is capable of detecting clinically relevant Sanders stages. While there have been previous attempts to determine bone age sonographically, we focused on comparability with the established SMS and TOCI classifications [13–17, 20].

Regarding demographic data, the majority of our patients were between 12 and 17 years of age. This is consistent with the fact described by Sanders et al. that the significant phase of the growth spurt occurs during adolescence and that, as a result, adolescent scoliosis, leg length discrepancies, and leg axis deformities become clinically prominent [1, 21]. SMS 3, which contained the most individuals, as well as SMS 7/8, TOCI 4/5, and TOCI 8 were found to be the most common, as these stages also represent phases during growth when physicians either indicate or omit surgical or conservative treatment options when diagnosing scoliosis or limb deformities. In addition, other growth stages, such as SMS 5, represent a rapid transition between SMS 4 and 6 [21]. The low number of patients in SMS 1 and 2 also results from the fact that there is rarely a justifiable indication for radiographs of the hand before the last growth spurt, since we know without further diagnostics that otherwise healthy children will still grow significantly. For these ethical reasons, we performed radiographs of the hand only when this had the potential to lead to a therapeutic decision.

The percentages of concordance in our data compare well with previous studies, e.g., Utczas et al. [15, 17]. These authors used a quantitative ultrasound-based device measuring acoustic parameters of the wrist [15]. Although it should be remembered that the trial population studied was almost ten times larger than ours, they used the complex Greulich and Pyle method as well as a specialized, not widely available ultrasound device [17].

Comparing *U* and *R*, we found average to good probabilities of an accurate attribution to the 8 Sanders and first 8 TOCI stages. Looking at the stages separately, we found the highest discrepancy in SMS 5, 6, and 8 as well as TOCI 3, 6, and 7. The highest agreements were found in SMS 3 and 7 with 71.7% and 66.0%, respectively, as well as TOCI 8 with at least 77.7%. It is noteworthy that these are the phases with the largest patient population. This might also have resulted in a better correlation with the radiologic result due to a better statistically relevant cohort. We speculate that the greater routine with these categories enabled physicians to diagnose these categories more easily, leading to lower statistical agreement in groups with fewer patients. In addition, it is probably easier to identify wide-open or fully closed growth plates, which increases the hit probability for Sanders 3, 7, and 8 and TOCI 8. We found a very small number of patients at TOCI 9 stage and therefore could not perform a statistically accurate evaluation.

The differences in SMS and TOCI scoring are well known in the literature and are also consistent with our results [8]. Hung et al. have previously described that TOCI 5 levels are more consistent with SMS 3 and TOCI 8 are more consistent with SMS 8 when radiological analysis is performed [8]. However, according to their data, both scoring systems are reliable tools for determining skeletal maturity.

In clinical practice, it is very important to have a simple and usable tool to identify the stages that entail therapeutic consequences. Therefore, we have grouped SMS 3 and 4 (in SMS category 2) and TOCI 5 and 6 (in TOCI category 2), where a residual growth of 10% can be expected and the initiation of brace therapy in case of scoliosis or temporary epiphysiodesis in case of limb deformities might be appropriate [3, 22]. We also combined SMS 7 and 8 (in SMS category 2) and TOCI 7 and 8 (in TOCI category 3), in which no therapeutically relevant growth occurs and the above therapy methods are omitted in most cases. In particular, for SMS categories 2 and 4, we found very acceptable transferability between U and radiography, with *P* = 76.7% [71.8%; 81.0%] and *P* = 79.7% [75.0%; 83.9%], respectively, making the assessment of SMS by *U* a reliable, readily available, rapid, radiation-free, and cost-effective tool with low burden on children.

Our intra- and interrater reliability for the radiological SMS and TOCI were good compared with other studies validating the SMS, so the data could be used as the basis for comparison with U [8, 23]. The intrarater reliability of U was slightly lower for both SMS and TOCI, but was within the limits for clear to strong agreement (Table 2). However, the interrater reliability of U for SMS and TOCI was weaker. In particular, for SMS, the agreement using Fleiss’ Kappa was weak (*k* = 0.381). Looking at the different concordances when comparing R and U devided by raters, there was a wide range between *P* = 70.4% and *P* = 33.0% (see Table 1).

Differences in interrater agreement are well known in ultrasound diagnostics in different medical fields compared to other imaging modalities and often depend on the level of experience of the examiner [24–27]. We have observed significant differences among the raters concerning concordance, but we have achieved very satisfying results in terms of intrarater reliability. This indicates that raters consistently scored at a high and reproducible level but interpreted the data systematically differently from their colleagues. Therefore, we hypothesize that providing more intensive training before conducting SMS or TOCI using US could lead to improved accuracy of the method.

Nevertheless, SMS can also be effectively determined by *U* by combining the clinically relevant stages SMS 3/4 and SMS 7/8. With somewhat higher intra- and interrater reliability, our data show advantage of radiographic determination of SMS compared with the use of *U*. However, because *U* is radiation-free, easy to perform, inexpensive, and rapid and involves very little psychological distress to the child, it may offer advantages in clinical practice.

## Data Availability

The data generated and used for the study is available upon request.
